# EU climate action through an energy poverty lens

**DOI:** 10.1038/s41598-023-32705-2

**Published:** 2023-04-13

**Authors:** Toon Vandyck, Nives Della Valle, Umed Temursho, Matthias Weitzel

**Affiliations:** 1 European Commission, Joint Research Centre (JRC), Calle Inca Garcilaso 3, 41092 Seville, Spain; 2 Department of Economics, KU Leuven, Naamsestraat 69, 3000 Leuven, Belgium; 3 European Commission, Joint Research Centre (JRC), Via E. Fermi 2749, 21027 Ispra, Italy; 4 IOpedia, Seville, Spain; 5 Institute of Public Policy and Administration, Graduate School of Development, University of Central Asia, Bishkek, Kyrgyz Republic

**Keywords:** Energy justice, Energy access, Energy and behaviour, Environmental social sciences, Climate-change policy, Energy and society, Psychology and behaviour, Socioeconomic scenarios

## Abstract

Carbon pricing can steer energy choices towards low-carbon fuels and foster energy conservation efforts. Simultaneously, higher fossil fuel prices may exacerbate energy poverty. A just portfolio of climate policies therefore requires a balanced instrument mix to jointly combat climate change and energy poverty. We review recent policy developments in the EU aimed at addressing energy poverty and the social implications of the climate neutrality transition. We then operationalise an affordability-based definition of energy poverty and numerically illustrate that recent EU climate policy proposals risk raising the number of energy poor when not accompanied with complementary measures, while alternative climate policy designs could lift more than 1 million households out of energy poverty through income-targeted revenue recycling schemes. While these schemes have low informational requirements and appear sufficient to avoid exacerbating energy poverty, the findings suggest that more tailored interventions are needed. Finally, we discuss how insights from behavioural economics and energy justice can help shape optimal policy packages and processes.

## Introduction

Meeting the 1.5 °C target of the Paris Agreement calls for ambitious climate action. To deliver on announced pledges, governments need to convert targets into comprehensive and coherent policy packages that can jointly achieve climate action (Sustainable Development Goal, SDG 13) and access to affordable and clean energy (SDG 7). Price-based instruments, such as carbon taxes or cap-and-trade systems, have been implemented in various countries, and feature prominently in ongoing policy proposals. For instance, the Fit-for-55 package that aims to deliver on the targets set out in the EU Green Deal proposes an extension of the EU Emission Trading System (EU ETS) to cover emissions from land transport and buildings. At the same time, price-based measures like carbon taxes risk being regressive when not accompanied by complementary measures, and could trigger concerns around fairness, as vulnerable citizens usually spend a higher share of their income on carbon-intensive goods to meet their basic needs^1^. Therefore, the public acceptance of such measures cannot be taken for granted^2^, and distributional objectives should be considered from the outset in policy design and communication^3^. Numerical studies suggest that the additional revenue of carbon pricing can be used to overcome the equity concerns (e.g.^4^). Often, quantified analyses take a stylized view on social aspects, for instance summarizing equity considerations through welfare impacts across income deciles. However, heterogeneity across households stems for a multitude of sources, and socio-economic challenges go beyond aggregated incomes^5^. Furthermore, recent evidence suggests that revenue recycling may not be a silver bullet when it comes to garnering public support^6^.

A broader conceptual framework may therefore prove valuable in fostering a just transition, which can be defined as “a fair and equitable process of moving towards a post-carbon society”^7^. Such a framework can provide policymakers with a lens to understand the nature of and solutions to the potential trade-offs in the energy transition^8^. Opening up a wider perspective based on concepts from social, environmental and energy justice can pave the way to address a more comprehensive range of social inequities that interact with the transition towards a low-carbon economy^9,10^. Of these broader social considerations, energy poverty is of particular concern in discussions around equitable climate policy. While the problem is rooted in distributional inequalities in terms of access to affordable energy services, effective solutions require elements from all components in the three-tenet energy justice framework: distributional, recognition and procedural injustice^11,12^. These considerations are key in Europe, where tackling energy poverty has recently emerged as a specific policy priority. In the current context of high energy and food prices^13,14^, designing climate policy to address rather than exacerbate social concerns is an opportunity for societal progress, as well as essential for the acceptability of ambitious climate action. The scientific community has called for action on energy poverty in the EU^15^, and academic research on energy poverty has been rapidly growing over the past 5–10 years^16^. While recent papers call for a stronger focus on fairness issues in integrated assessment modelling^17–19^, energy poverty has received relatively little attention in quantified scenario analysis thus far. By overlooking the consequences for energy poverty, recent research risks conveying the overly simplified message that optimal policy design consists of carbon pricing combined with uniform lump sum transfers to households.

In this study, we seek to advance the debate on energy poverty by further aligning ongoing policy developments, quantified pathway analysis and recent insights from the broader academic literature. First, we take stock of the recent EU climate and energy policy answers to the challenge of energy poverty. Second, we operationalise a definition of energy poverty that enables its quantification in model-based climate policy pathways for the EU. A numerical application complements the assessment of the European Commission’s recent climate policy proposals with the inclusion of targeted revenue recycling mechanisms and reveals the corresponding energy poverty implications. Finally, we discuss how insights from behavioural economics and energy justice can be leveraged to inform the design of a justice-aware climate and energy policy package.

## Energy poverty in EU policy

**Figure 1:**
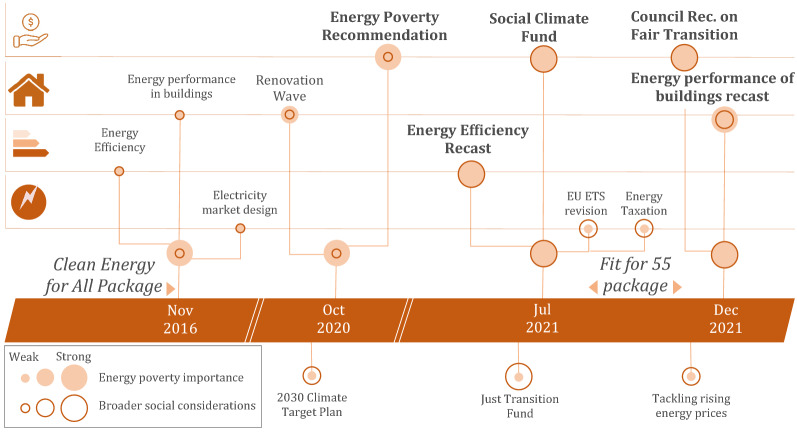
Timeline of recent EU climate and energy policy initiatives and their focus on energy poverty and social aspects. For an explanation of symbol scaling, see “Methods”.

A review of recent EU climate and energy policy proposals reveals four layers of action that relate to energy poverty (Fig. 1): initiatives directly related to energy poverty and broader social aspects; energy performance of buildings; energy efficiency measures; and related climate and energy policies. Figure 1 furthermore illustrates that the presence of energy poverty in the policy narrative is stronger in more recent initiatives, suggesting a growing importance and mainstreaming of energy poverty considerations into related policy initiatives (see “Methods”).

The *Just Transition Fund* and the *Social Climate Fund* earmark financial means to resolve spatial inequities and protect vulnerable households, respectively, with the latter also strengthening governance aspects by asking Member States to draft *Social Climate Plans* in which they outline the foreseen measures and investments. These Social Climate Plans complement the *National Energy and Climate* Plans, which originate in the *Clean Energy for All Europeans package* and require a stocktake of energy poverty, along with the corresponding formulation of objectives to reduce energy poverty^20^. While we focus here on the interaction between climate action and energy poverty at the EU policy level, governance aspects are important as many of the policy levers to address energy poverty are at the national or subnational level. In that respect, the proposed *Council Recommendation on Ensuring a Fair Transition towards climate neutrality* provides further guidance on accompanying measures regarding the transition on the labour market and associated skill needs, fair tax-benefit systems, affordable housing, policy coordination, stakeholder engagement, harmonisation and strengthening of the evidence base and optimal use of public and private funding.

In addition to policy mainstreaming, financial support, governance and guidance, a number of support bodies have been created to facilitate knowledge-sharing. Initiatives like the *Energy Poverty Advisory Hub* (formerly the EU Energy Poverty Observatory), the *Citizens' Energy Forum* and the *Energy Poverty and Vulnerable Consumers Coordination Group* provide platforms to collect expertise, gather stakeholders and strengthen collaboration to facilitate tackling energy poverty by national, regional and local governments.

## Assessing energy poverty through policy modelling

Comparing potential outcomes of proposed policy options enables a science-based societal dialogue. In this process, model-based, quantified emission pathways can inform policy design by revealing synergies and trade-offs ex ante^21,22^. Recent work^23^, for instance, illustrates how climate policy interacts with the achievement of other sustainable development goals. While revealing various synergies, the results also indicate potential trade-offs between climate action and other developmental indicators such as poverty, due to rising energy and food prices. The authors propose additional interventions to align climate and development agendas, including redistributive transfers within and across countries, as well as measures to improve energy access and change dietary patterns. Other recent studies^19,24^ similarly caution that climate policy without revenue recycling (and ignoring the distribution of avoided damages) risks exacerbating poverty, while highlighting that progressive revenue redistribution would more than offset this effect. Energy poverty has received less attention in the literature on sustainable energy pathways. The existing work focuses on access to energy in a global context, and suggests that climate action (SDG13) does not guarantee universal energy access (SDG7), although both can be achieved simultaneously through directed policy intervention^25,26^.

**Figure 2:**
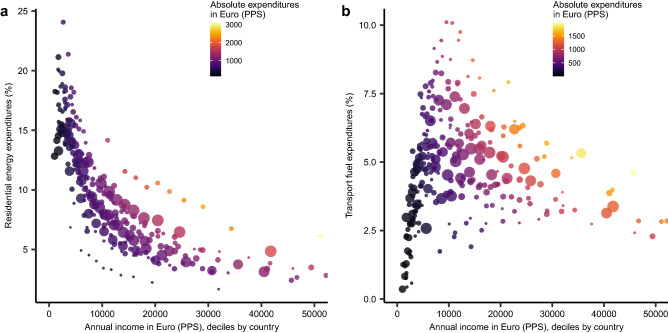
Expenditure shares of residential and transport energy by income group and country. Share in total expenditures of (**a**), residential energy (CP045), and (**b**) fuels and lubricants for personal transport equipment (CP0722). Lines connect the income deciles within a country. Incomes and absolute expenditures are annual and expressed in purchasing power standards. Truncated horizontal axis does not show the tenth decile for Luxembourg. PPS, Purchasing Power Standard. Source: Household Budget Survey 2015, Eurostat.

Household-level survey-based descriptive statistics provide a glimpse of one of the multiple dimensions of energy poverty^5,26–29^, as the share of residential energy in total consumption expenditures generally declines with income (Fig. 2a). Few countries (Poland, Romania, Lithuania) deviate slightly from this pattern, where solid fuels and derived heat play a relatively strong role for household heating. In the EU, a high-income region with relatively widespread access to modern energy compared to other parts of the world, concerns about affordability prevail over accessibility in the energy poverty debate^27^. Correspondingly, we operationalise a metric of energy poverty count based on affordability, categorizing a household as energy poor when expenditures on residential energy exceed a threshold share, which we set here at 10% in line with earlier work^28^, and when household income lies below 60% of median income in the corresponding country (see “Methods”). The described metric for energy poverty enables an ex ante model-based assessment (see “Methods”) of the impact of recent climate policy proposals in the EU, in particular ratcheting up the 2030 target to 55% reduction in greenhouse gases relative to 1990 levels (*ClimPol*), and related revenue recycling options (*ClimPol* + *Tr*)^29,30^.

Private transport fuel expenditures typically show an inverse U-shaped relation with income (Fig. 2b) and are not considered here, although they may give rise to horizontal equity issues—impact differentiation within income groups—that warrant further attention^31,32^. The pattern may be explained by low travel demand or by low car ownership rates at the bottom end of the income distribution, as public transport (e.g. bus) costs—including time cost—are not shown in the figure. Moving up the income scale, the figure suggests that, initially, private transport fuel has an income elasticity larger than one, with budget shares increasing with income. After some income threshold, the private transport fuel expenditure share appears to decline again, as private transport demand saturates as income increases further (absolute expenditures are not declining as shown by the colouring in Fig. 2b). Other elements may further contribute to this pattern, such as fuel-efficiency of cars, residence in urban centres with bike or metro use, or additional expenditure on transport services (taxi, plane). These observations indicate that a study of transport poverty would need to account for the (spatial) interactions between the choice of residence, travel time and mode choice.

**Figure 3:**
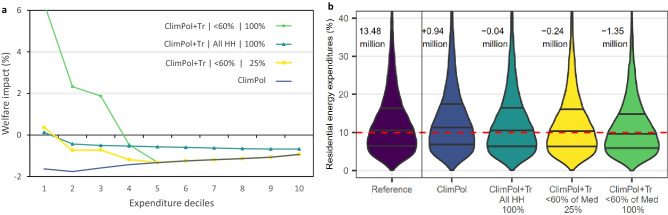
Distributional and energy poverty implications of strengthening climate action. (**a**) Welfare impacts across different expenditure deciles and scenarios (see main text). The welfare metric used is compensating variation expressed as a percent of total expenditures. (**b**) The violin plots show the distribution of the share of residential energy expenditures in total income for the households in the EU below the poverty line of 60% of median income in the respective country. Dashed red line indicates the cut-off point of 10% used to calculate the energy poverty indicator. Numbers depicted in the upper part of the chart indicate the change in the number of households in the EU in energy poverty due to climate policy and revenue recycling schemes relative to the Reference value of 13.48 million households in energy poverty. *Reference* refers to the situation under current policies; *ClimPol* introduces additional climate policy including the extension of EU ETS to buildings; *ClimPol* + *Tr* considers additional climate policy as well as lump-sum transfer recycling of (100% or 25% of) the additional tax revenue to (all or poor) households. Source: Own analysis based on the JRC-GEM-E3 model and the EU Household Budget Survey.

The results of the pathway analysis (Fig. 3a) confirm earlier findings that price-based climate policies may lead to regressive outcomes when not accompanied with compensating measures (*ClimPol*), while full revenue recycling through uniform lump-sum transfers to all households can bring about a progressive distributional pattern (*ClimPol* + *Tr|All HH|100%*). In addition to existing works, we show here that already a partial (25% of all additional revenue) revenue recycling through lump sum transfers targeted to poor households (below 60% of median income) can bring average net benefits to the 10% poorest households (*ClimPol* + *Tr|*< *60%|25%*), while leaving a substantial amount of funds available for other supportive measures (for which impacts are not considered in Fig. 3). The results furthermore suggest that channelling all additional revenues to the households with income below 60% of the median (*ClimPol* + *Tr|*< *60%|100%*) can generate substantial welfare gains (< 6%) for the bottom 10%.

The climate policy scenario assessment furthermore quantifies the potential implications of enhanced climate action for energy poverty in the EU. Figure 3b visualizes the distribution (with horizontal lines representing median and 25th and 75th percentiles) of residential energy expenditure shares among the population with household incomes below 60% of the median in the country, for the current-policy *Reference* and the same scenarios as shown in Fig. 3a. The advantage of this representation is that it shows also the tails of the distribution, with a small fraction of households spending more than one third of their budget on expenses to heat their home. According to the definition we adopt here, households are classified as energy poor when their place in the distribution is above the dashed red line of 10%. Without compensatory measures, the climate policy package studied here (*ClimPol*) could push an additional 1 million households into energy poverty by raising residential energy expenditures. Recycling all of the additional carbon pricing revenue via uniform lump sum transfers to all households quantitatively offsets this risk, with the number of households in energy poverty remaining roughly on par with the status quo. Non-targeted transfers therefore leave the underlying problem of energy poverty unresolved. Furthermore, the results indicate that a targeted use of (25% or 100% of) proceeds of additional pricing measures via means-tested uniform lump-sum transfers can reduce the count of households facing energy poverty. Targeted transfers to households below the poverty line thus represent a more effective means to support low-income groups. At the same time, these generic (targeted) transfers are unlikely to be sufficient to compensate hardship cases, and lack precision to resolve energy poverty effectively. While the overall distribution of residential energy expenditure shares shifts downwards, Fig. 3b illustrates that the changes to the shape of the distribution are minor and the upper tail is not targeted by the recycling scheme considered here. Our findings therefore suggest that although simple revenue recycling schemes as considered in recent literature^19,24^ may generate progressive impact patterns, a tailored set of policy interventions will be required to tackle related social issues such as energy poverty^15^.

## Addressing energy poverty through lessons from a behavioural economic perspective on energy justice

To operationalise distributive justice, a tailored set of measures targeted to the energy poor would thus be ideal. The design and implementation of this package, however, is not devoid of challenges. A behavioural-economic perspective on energy justice can help unpack some of the challenges involved and help frame solutions. Beyond fiscal measures, a policy package can build on distributive justice theories^9^. This implies evaluating how social goods (like energy) and ills (like policy costs) are distributed across the society^33^, and how such distributions are distant from proper ones, such as those reflecting the need principles^9^. As an example, energy poverty is seen as a violation of a basic universal right (i.e. physical security), when considering that a certain set of minimal energy services is a key condition to enable everyone to live in a clean and safe environment^9^.

While policy makers play a key role in the process leading to a just energy system, in reality its operationalisation is modulated by all the involved actors, above all citizens^34^. First, citizens’ preferences over the distributions of costs and benefits associated with a certain policy package are heterogeneous^35^. If the policy package does not sufficiently and accurately factor in this heterogeneity, it will unlikely receive public support and be hardly applicable^1^ (acceptability problem). Second, the unequal distribution of energy services across the society is underpinned by a lack of recognition and procedural justices^12^. If heterogeneous energy needs are not sufficiently recognised in policy design, transfers could be misallocated, and energy poverty perpetuated^36^ (targeting problem). At the same time, if vulnerable actors are kept as passive receivers of interventions, policy decisions will only partially represent and meet their energy needs^37^ (responsibility problem). Therefore, while complex societal challenges can be addressed better if involved actors act collectively^38^, this capacity to act needs to be enhanced^39^, considering individuals’ surrounding context and heterogeneous motivational and cognitive structures^40^. Below, we illustrate how the evidence-based insights offered by the field of behavioural economics can be used to inform the design of interventions enabling citizens to become co-enablers of just climate policy packages.

### Acceptability problem

It is not guaranteed that a fair policy package identified by policy makers, like a carbon pricing combined with a financial mechanism targeted to the energy poor, will automatically receive public support^41^. A growing experimental economic literature shows that individuals care not only about outcomes (like benefits and costs), but also about how these are distributed^42^. These distributive justice concerns are also heterogeneous across individuals^43–46^. As a result, a certain use of carbon tax revenues might not receive public support if individuals perceive it as unfair^47,48^. At the same time, these perceptions can often be biased and lead to costs for society.

First, experimental studies have proven that distributive preferences intersect with bounded rationality problems^43^. In particular, citizens assess the costs and benefits associated with a policy under limited cognitive capacity^49^. Second, extensive experimental evidence has proven that individuals use fairness principles (like need or accountability) in a self-interested and self-serving way when interpreting the fairness of a policy^43^. As an example, when individuals want to avoid costs from policy compensation measures, they might alter their beliefs that not compensating low-income households is fair. In particular, to avoid cognitive dissonance (i.e. it is too cognitively costly to think they are selfish), individuals self-deceive about what is fair^35^, e.g., by altering their beliefs that vulnerable groups’ adverse real-life conditions are due to poor choices or lack of effort rather than to factors beyond their control^45,50^.

Consequently, to enable individuals to evaluate the fairness of a certain policy package in a less biased way, a climate package can be augmented with an instrument that factors in empirical evidence on human behaviour^51^, like a nudge that implements a change in the framing of the policy communication^49^. As an example, policymakers can broaden public support^52^ by leveraging the evidence that exposing citizens to different types of information on the source of inequality is an effective measure for reducing polarization in self-serving views of fairness^53^.

### Targeting problem

A policy package that has potential to attain both climate change and energy poverty objectives, like carbon pricing combined with targeted compensatory measures, shall above all be able to effectively reach the target group, i.e. the energy poor. However, policy makers are usually unable to fully detect the multi-dimensional attributes underlying energy poverty^5^. Consequently, policy efforts might risk failing to effectively address energy poverty due to exclusion of target groups from dedicated measures through misrecognition and imprecise targeting^54^.

In this context, advances in technology (e.g. smart meters) hold a great promise for addressing these informational barriers^55^. As an example, they can enable revealing hidden energy needs, like those of who are forced to restrain (and thus hide from the traditional energy-expenditure based indicators, as the one we used earlier) their energy consumption to prioritise other basic goods^27,56^.

At the same time, energy monitoring enabled by smart meters might yield unintended consequences for citizens, who might be *sludged* to agree that their data can be used for commercial purposes^57,58^. More particularly, malevolent powerful actors can exploit individuals’ tendency to accept the status quo by designing unethical privacy default options^59^. Consequently, policymakers can augment the climate policy package with additional instruments offered by behavioural economics^60^. As an example, they could devise a boosting intervention^61,62^, such as in the form of a digital literacy training, which has been proven to empower citizens’ competencies of reasoning and resilience to manipulation^63^.

To counter the limited access to energy poverty data, policy makers may also leverage the same energy poor’s inputs on their experiences^34^. However, sharing this type of information is often associated with stigma^64^. As a result, policymakers can augment the climate policy package by designing a behavioural instrument in the form of a nudge, informing citizens that similar peers are already providing their inputs on their needs and problems. This norm-based nudge might provide them a motivation to do so as well^34^, as extensively proven for other types of behaviours, like pro-environmental^65^ and cooperative^66^ ones.

### Responsibility problem

The process towards a more equitable and inclusive energy transition is “about more than just technological and political change, (…) it also involves significant social and behavioural transformations that question historical narratives and challenge accepted understandings of democracy and economics”(^67^, p. 2). Such profound transformations are accompanied by the emergence of new social roles and responsibilities^68^. The role of *energy citizen* is increasingly gaining attention in the debate on how to operationalise energy justice in the energy system^69^. This role is linked to an active form of participation which goes beyond passive intervention acceptance^70^ and is manifested in an active participation to the relevant energy decision-making processes^71^, taking *responsibility* for energy production and consumption^72^.

Promoting this role of energy citizen is especially crucial when looking at the energy poor. First, because following financial measures, individuals might increase their consumption over energy services or start consuming previously unaffordable energy services, which are associated with emissions^73^. As a result, citizens could be encouraged to take *responsibility* for energy consumption through a behavioural intervention, like a nudge that communicates the environmental impacts of their energy consumption^74^. This type of nudges, which has been proven to be effective at promoting pro-environmental behaviour change^75^, might enable citizens to consume energy in a more responsible way. Importantly, trusted actors, like one-stop-shops, would enable local and regional authorities to acknowledge better the shared responsibility to overcome principal-agent problems, such as the tenant-landlord split incentives^76^.

Second, if the energy transition has also to seek fairness and equity, then those who directly experience those injustices, like the energy poor, have to be enabled to have a say on how to achieve these goals^37^. In this case, the application of behavioural insights could help policymakers elevate citizens from being passive policy recipients to become capable and knowledgeable thinkers^77,78^. One way to achieve this would be bringing citizens into policy development through *thinks* and *nudges plus*. *Thinks* are broadly defined as deliberative interventions^77,79^, where citizens can get involved by reflecting on a problem and having their say on the potential solutions, such as through citizens’ juries, citizens’ assemblies and participatory budgeting^77^. *Nudges plus* add the deliberative element of thinks to a nudge and can result from a co-design process that involves citizens’ and local policymakers’ expertise^80^. These interventions, which have been proven to promote individual agency while ensuring that individuals are saved from the cognitive burden of deliberating^39^, might enable vulnerable citizens to get their specific energy needs better represented in and met by policy decisions. Initiatives, such as the *Energy Poverty Advisory Hub*, energy cooperatives^81^ and trusted intermediaries^82^ can facilitate this process across local and regional authorities in the EU.

## Concluding remarks

Achieving the goals of the Paris Agreement requires accelerating the transition to a low-carbon energy future. The associated transition risk includes the reinforcement of existing inequalities and the creation of new injustices, including energy poverty. Climate policies, like carbon pricing, can be regressive, exacerbating asymmetries in the distributions of assets (e.g. energy costs, income) necessary to meet energy needs. At the same time, a well-managed structural transition to climate neutrality offers opportunities to address related societal issues such as energy poverty.

In this study, we quantified the distributive implications of carbon pricing combined with different measures and found that low-income targeted revenue recycling can bring progressive outcomes while mitigating energy poverty. However, the results also indicate that generic transfer schemes are insufficient and inefficient to tackle the problem of energy poverty head-on, which calls for dedicated policy interventions. We then complemented the numerical assessment with a behavioural economic analysis of energy justice to guide justice-considerate policy design. Behavioural economic insights can be used to address some of the problems that might hinder policy targeting and policy acceptance. These insights can also be used to address the recognition and procedural injustices underlying energy poverty, by providing policymakers with ways through which engage the energy poor as energy citizens. Finally, these insights are based on empirical and experimental evidence offered by related studies. As such, they can provide a direction to follow in an evidence-based approach, where the proposed behavioural interventions are validated in the specific context, such as through the use of experiments on the target population^83^.

We adopted one of the dominant energy justice frameworks originated from works in developed countries^84^. We also referred to one possible conceptualisation of energy poverty, i.e. in terms of energy service affordability, which might not capture the importance of energy services for socio-economic development, wellbeing and quality of life, as it is for developing countries^85^. An avenue for future research would be to expand this conceptualisation of energy justice with the tenet of restorative justice and the understanding of energy poverty using the capabilities space, to account also for the specificities of developing countries. Future model-based assessments could further explore the quantitative impacts of a broader and tailored set of interventions, including fiscal and behavioural components.

Finally, since the transition to an equitable low-carbon society requires a better recognition of those groups who are simultaneously at risk of experiencing energy and transport poverty^32^, a future expansion of this work will be to study the distributive implications of climate policy also accounting for the links between energy and transport poverty. This would require a broader framework that accounts for spatial interactions, as transport and housing choices are closely linked.

## Methods

### Review of policy initiatives

The EU climate and energy policy initiatives displayed in Fig. 1 mention the term “energy poverty” at least once. The size of fonts and of the colour-filled circle is scaled to the number of appearances of the term “energy poverty” in the corresponding policy document, while the circle outline is scaled to a summed count of appearances of the expressions “just transition”, “fair transition”, “vulnerable” and “vulnerability”, indicating the importance of broader social considerations in the respective policy initiative and the corresponding narrative (Table 1). We acknowledge that this overview is not an exhaustive list of all relevant policy developments, e.g. social policy initiatives, the Effort Sharing Regulation, the October 2021 toolbox to tackle rising energy prices or the RePowerEU Plan may additionally affect distributional outcomes and energy poverty indirectly.

**Table 1:** Overview of selected policy initiatives.

Date	Documentation	Policy initiative	Package	Word count				
				"Energy poverty"	Combined social	"Just transition"	"Fair transition"	"Vulnerable"; "Vulnerability"
Nov-16	COM(2016) 765	Energy performance in buildings	CE4A	8	1	0	0	1
Nov-16	COM(2016) 761	Energy efficiency	CE4A	8	1	0	0	1
Nov-16	COM(2016) 861	Electricity market design	CE4A	9	4	0	0	4
Sep-20	COM(2020) 562	2030 Climate Target Plan		2	13	10	1	2
Oct-20	C(2020) 9600	Recommendation on Energy Poverty		35	8	0	2	6
Oct-20	COM(2020) 662	Renovation wave		16	6	1	0	5
Jun-21	REG 2021/1056	Reg. establishing the Just Transition Fund		3	46	44	0	2
Jul-21	COM(2021) 568	Social Climate Fund	FF55	27	96	13	0	83
Jul-21	COM(2021) 551	Revision of EU ETS	FF55	4	13	6	0	7
Jul-21	COM(2021) 558	Energy Efficiency Recast	FF55	81	61	2	3	56
Jul-21	COM(2021) 563	Energy Taxation Directive	FF55	2	18	2	0	16
Oct-21	COM(2021) 660	Tackling rising energy prices		9	20	1	0	19
Dec-21	COM(2021) 801	Proposed Council Recom. on Fair Transition	FF55	36	119	28	38	53
Dec-21	COM(2021) 802	Energy Performance of Buildings Recast	FF55	26	23	4	2	17

*CE4A* Clean Energy for All Europeans package, *FF55* Fit for 55 package.

### Energy poverty metric

We use the following equation to calculate the share of residential energy expenditures ${REE}_{i}$ in total expenditures ${E}_{i}$ of household $i$ in the scenarios:

$$\frac{{REE}_{i}}{{E}_{i}}=\frac{{REE}_{i}^{0}\left(1+\Delta p\right)-{T}_{1}}{{E}_{i}^{0}+{REE}_{i}^{0}*\Delta p+{T}_{2}}$$

The superscript 0 indicates benchmark values before policy reform, $\Delta p$ represents the calculated relative change in expenditure compared to benchmark levels, and ${T}_{1}$ and ${T}_{2}$ are monetary transfers of additional public revenue. Three conditions apply to the parameters in this equation:

$$Cond. 1: {T}_{1}\le {REE}_{i}^{0}*\Delta p*\left(1-\frac{{REE}_{i}^{0}}{{E}_{i}^{0}}\right)$$

$$Cond. 2: {\Delta p<0\underset{}{\Rightarrow }T}_{1}=0$$

$$Cond. 3: {T}_{1}+{T}_{2}=T$$

The intuition behind Condition 1 is to use the transfer ${T}_{1}$ to restore expenditure share of residential energy to benchmark levels. The term $\left(1-\frac{{REE}_{i}^{0}}{{E}_{i}^{0}}\right)$ is added because ${REE}_{i}^{0}*\Delta p$ also adds to the denominator in the formula. Additional transfers ${T}_{2}$ are then added in the denominator, implicitly assuming that this money is spent on goods and services other than residential energy. Condition 2 prevents this mechanism to apply in case of decreased residential energy expenditures. Condition 3 ensures government budget neutrality by stating that the transfers equal the total additional revenue $T$.

We focus here on operationalising one indicator, while we acknowledge the wider debate about energy poverty metrics^86^ and point to a number of advantages and caveats of the metric adopted here. One advantage is the intuitive nature of the metric, as a household’s energy poverty status is unchanged when monetary transfers exactly offset increased energy expenditures. Furthermore, this metric relies on expenditure data and therefore can be used in ex ante assessments, in contrast with subjective answers to survey questions that relate to a household’s ability to keep the home warm. In addition, this metric explicitly acknowledges the affordability dimension of energy poverty. At the same time, a number of caveats apply. The metric is insensitive to whatever happens above the 10% threshold and therefore disregards the distribution of energy expenditures. Relying on one summary measures therefore offers only incomplete information. Furthermore, this quantification ignores hidden energy poverty, as it does not pick up households that cannot spend on energy (and related conditions in the house, e.g. comfort) because facing other urgent expenses, such as medical and health-related expenses. Finally, the metric presented is not multidimensional, ignores energy accessibility and reliability, and takes no account of related and compound effects such as transport poverty.

### Model-based assessment

The quantitative analysis builds on a modelling framework that combines a bottom-up energy system model, a multi-sector economic model, and a household-level microsimulation tool. An extensive description of the scenario implementation in the multi-sector economic model JRC-GEM-E3 can be found in Weitzel et al.^87^. This model is a computable general equilibrium (CGE) model, for which the mathematical statements are detailed in Capros et al.^88^. The microsimulation is based on the 2010 Household Budget Survey as harmonised by Eurostat, which implies that 25 EU countries are covered in the analysis here (not Austria and the Netherlands). A more elaborate explanation of the coupling between JRC-GEM-E3 and household-level data is provided by Temursho et al.^89^.

The scenario we study is a combination of price and non-price (e.g. standards) measures corresponding with the MIX scenario as assessed in the policy context^30^, which includes an extension of the EU Emission Trading System to transport and buildings, effectively putting a price on household emissions. Importantly, this scenario represents a ratcheting up of the 2030 climate policy target to a net 55% reduction in greenhouse gas emissions relative to 1990 levels. Revenue recycling is implemented within EU countries, ignoring the possibility of monetary transfers across countries, and achieving budget neutrality through uniform transfers per equivalised household (using modified OECD equivalence scales).

## Data Availability

The data that support the findings of this study are available from EUROSTAT but restrictions apply to the availability of these data, which were used under license for the current study, and so are not publicly available. Data are however available from the corresponding author upon reasonable request and with permission of EUROSTAT.
